# DNase I alleviates renal inflammatory injury in MRL/lpr mice by inhibiting NETs formation

**DOI:** 10.3389/fimmu.2025.1656069

**Published:** 2025-10-23

**Authors:** Manling Zhang, Xinran Xie, Gula Da, Hongbin Li, Yong Jin

**Affiliations:** ^1^ Department of Rheumatology and Immunology, The Affiliated Hospital of Inner Mongolia Medical University, Hohhot,, China; ^2^ Inner Mongolia Key Laboratory of Pathogenesis and Immunodiagnosis of Rheumatic and Immunological Diseases, The Affiliated Hospital of Inner Mongolia Medical University, Hohhot, China; ^3^ School of Basic Medicine Sciences, Inner Mongolia Medical University, Hohhot, China

**Keywords:** DNase I, NETs, renal inflammatory injury, MRL/lpr, lupus nephritis

## Abstract

**Background:**

Lupus nephritis (LN) is one of the most common complications of systemic lupus erythematosus (SLE) and represents a frequent and potentially life-threatening clinical condition. The pathogenesis of LN involves multiple immune cell types. Notably, neutrophil extracellular traps (NETs) formation has been closely associated with renal inflammatory injury. However, the underlying pathophysiological mechanisms remain incompletely understood.

**Methods:**

We administered DNase I to MRL/lpr mice, monitored signs and renal pathology, quantified gene expression levels, and conducted flow cytometry and RNA-seq analysis. The expression levels of NETs molecular markers and key genes involved in relevant molecular pathways were assessed in both an *in vitro* cell model treated with PMA and DNase I, as well as in peripheral blood neutrophils from SLE patients, followed by correlation analysis.

**Results:**

Following DNase I treatment, the lupus-related manifestations, renal pathology, and renal function were significantly improved in the LN mouse model. The expression levels of MPO and CitH3 were reduced, and the expression of inflammatory damage molecules, including IL-1β, TNF-α, and Kim1, was down-regulated. RNA-seq analysis revealed that the neutrophil and T cell activation and chemotaxis pathways were suppressed, and the infiltration of cytotoxic immune cells in the kidneys was decreased in the DNase I-treated group compared to MRL/lpr mice. In an *in vitro* model of PMA-induced neutrophil activation, the addition of DNase I inhibited the expression of MPO and CitH3 and down-regulated the expression of inflammatory signaling molecules (TLR4, MYD88, and HMGB1), chemotactic molecule CCL2, and the key molecule of NETs formation, PADI4. Furthermore, the critical molecules PADI4, HMGB1, TLR4, and MYD88 were significantly upregulated in peripheral blood neutrophils from LN patients, and their expression levels in the kidneys of MRL/lpr mice increased in a time-dependent manner.

**Conclusions:**

DNase I alleviates renal inflammatory injury by inhibiting the NETs/TLR4/MYD88 cell signaling axis, reducing the formation of NETs and the infiltration of immune inflammatory cells such as T cells and macrophages. These findings may provide a novel clinical prevention and treatment strategy for LN.

## 1 Introduction

Lupus nephritis (LN) represents a prevalent and severe complication of systemic lupus erythematosus (SLE), serving as a major cause of mortality among patients. It predominantly affects women and individuals of Asian, African, and Hispanic descent (1). Despite significant advancements in immunosuppressive agents and other therapeutic modalities over the past decades, the complete remission rate remains below 50%, with a 2-year recurrence rate reaching up to 30%. Additionally, approximately 30% of patients progress to chronic renal insufficiency (2). Given the intricate pathogenesis of LN, particularly involving the activation of diverse immune cells and their mediation of inflammatory damage to renal parenchymal cells (3), elucidating the mechanisms of immune-mediated injury in LN and developing targeted therapies remain critical challenges for researchers and clinicians.

As the most abundant innate immune cells in the blood, neutrophils exert a critical regulatory function in the pathogenesis of LN (4–6). They contribute to renal tissue damage, increased urine protein levels, and renal function decline through infiltration (7). Recent studies indicate that targeted intervention of neutrophil aggregation can significantly ameliorate kidney injury (8). In addition to directly damaging renal intrinsic cells through phagocytosis, neutrophils can induce tissue necrosis and inflammatory self-amplifying cycles in the kidney by releasing extracellular traps composed of chromatin fibers carrying immunostimulatory molecules, as well as various enzymes and proteins from the cytoplasm and neutrophil granules, known as neutrophil extracellular traps (NETs) (9–11). The process by which NETs are formed is referred to as NETosis., involves the activation of multiple protein kinases. During NETosis, the release of nuclear DNA from neutrophils entails several critical steps: i) Chromatin decondensation: The citrullination of arginine residues on histone H3, catalyzed by peptidyl-arginine deiminase 4 (PADI4), represents a pivotal initiating event that drives chromatin decondensation. This modification weakens the interaction between histones and DNA (12). ii) Nuclear envelope rupture: Disruption of the nuclear envelope is mediated by phosphorylation of lamins. Phosphorylation of lamin B by protein kinase Cα (PKCα) has been shown to directly compromise nuclear envelope integrity (13). Additionally, phosphorylation of lamin A/C by cyclin-dependent kinases CDK4/6 also promotes nuclear membrane disintegration, enabling chromatin translocation into the cytosol (14). iii) Plasma membrane breakdown: The release of decondensed chromatin complexed with cytotoxic proteins into the extracellular space requires plasma membrane disruption. This final step is facilitated by cytoskeletal remodeling, particularly the disassembly of the actin cytoskeleton, which plays a crucial role in the assembly and release of NETs (15, 16).

Through renal biopsy of LN patients, it was confirmed that NETs were present in the glomerular region of inflammatory injury, and the degree of NETs infiltration was positively correlated with the pathological manifestations of LN (6). Studies have reported that NETs can induce a resident inflammatory response in the kidneys of patients with active LN (17). Mechanistic investigations revealed that NETs expose the histone-DNA complex to activate Toll-like receptors (TLRs) *in vivo*, thereby promoting type I interferon synthesis by plasmacytoid dendritic cells (18, 19). In the inflammatory lesion psoriasis, NETs can activate the Toll-like receptor 4 (TLR4) signaling pathway to amplify skin inflammation, and further recruiting neutrophils to the skin, thus exacerbating skin inflammatory injury (20). In models of liver inflammatory injury, PADI 4 inhibitors or DNase I treatment can significantly protect hepatocytes and mitigate the inflammatory response following liver ischemia-reperfusion (I/R) injury by targeting the inhibition or degradation of NETs (21). DNase I has also been shown to significantly reduce NETs levels, decrease inflammatory cell infiltration into tissues, and alleviate organ inflammatory damage in the kidneys of rats with vasculitis (22).

In our previous study, we integrated bioinformatics analysis of PBMCs with kidney transcriptomics data from LN patients sourced from the GEO database to identify and validate core genes associated with neutrophil activation and closely linked to the pathogenesis of LN. Dysregulated expression of these core genes was significantly correlated with the formation of NETs resulting from neutrophil activation. Furthermore, we demonstrated that DNase I effectively targeted and degraded NETs in the kidneys of LN mouse models, leading to a significant improvement in renal function (23). However, the precise molecular mechanism by which DNase I mitigates renal inflammatory injury in LN through targeting NETs remains to be fully elucidated. To address this mechanistic question, we conducted RNA-seq analysis on kidney tissues from C57 mice, MRL/lpr mice, and MRL/lpr mice treated with DNase I. Furthermore, comprehensive molecular and experimental investigations were performed using both *in vitro* cell models and the *in vivo* MRL/lpr mouse LN model. These analyses, employing a range of molecular biological techniques, elucidated the specific mechanism by which DNase I promotes targeted degradation of NETs, suppresses NET-induced neutrophil activation, reduces infiltration of pro-inflammatory immune cells, and mitigates renal inflammatory injury. Specifically, NETs exacerbate renal inflammation through activation of the NETs/TLR4/MYD88 signaling axis, leading to recruitment of T cells, macrophages, and other inflammatory immune cells. DNase I interrupts this inflammatory cascade by downregulating the expression of key signaling molecules, including TLR4 and MYD88, thereby disrupting the NET-mediated injury loop. These findings offer novel insights and potential therapeutic targets for the future treatment of LN. Additionally, several critical molecules—PADI4, HMGB1, TLR4, and MYD88—were identified as being closely associated with NETs formation and function, suggesting their potential utility as molecular biomarkers for NETs-mediated renal injury in LN.

## 2 Methods

### 2.1 Mice and experimental treatment

The animal experiments in this study were conducted in strict compliance with the experimental protocol approved by the Medical Ethics Committee of the Affiliated Hospital of Inner Mongolia Medical University (ethical approval number: YKD202402089). Female MRL/lpr mice aged 6–8 weeks were procured from Spefu (Beijing) Biotechnology Co., Ltd., and female C57BL/6J mice aged 8 weeks were obtained from Beijing Weitong Lihua Laboratory Animal Technology Co., Ltd. In this study, a total of 25 MRL/lpr mice were designated as the experimental group, while 20 C57BL/6J mice served as the control group. The experimental groups were subdivided into model groups at 15, 17, 19, and 21 weeks of age, as well as DNase I treatment groups. The control groups were similarly divided into subgroups corresponding to 15, 17, 19, and 21 weeks of age. Considering that MRL/lpr mice begin to exhibit lupus-like skin manifestations at 17 weeks of age, mice in the DNase I treatment group received daily intraperitoneal injections of DNase I (11284932001, Roche) starting at 17 weeks until they were euthanized at 21 weeks. Mice in the 21-week MRL/lpr model group and the normal control group were handled according to previously described protocols (23).

### 2.2 Collection and biochemical analysis of serum and urine samples

In the 21-week model group, the DNase I treatment group, and the 21-week control group, serum and urine samples were collected at 21 weeks, ensuring a consistent time of day (9:00-11:00 am). The concentration of serum anti-dsDNA (5120, ADI) was quantified using an ELISA assay. The urine protein concentration was determined by the Coomassie Brilliant Blue (CBB) method, while the serum creatinine concentration was measured using a creatine enzyme-coupled with creatine alkaline oxidase method. Serum urea nitrogen concentration was assessed via the urease method. All reagent kits were procured from Nanjing Jiancheng Bioengineering Institute (C035-2-1, C011-2-1, C013-2-1).

### 2.3 Histological examination

Kidney tissues were fixed, embedded in paraffin, and sectioned at a thickness of 2 μm. Subsequently, the sections underwent HE staining, PAS staining, Masson staining, and were processed according to standard histological protocols for evaluation. Positive staining areas were quantified by measuring five randomly selected fields under a light microscope (Leica). The pathological changes in renal tissue were semi-quantitatively assessed according to the previously published literature (23). Collagen-positive areas were further quantified using ImageJ software (National Institutes of Health).

### 2.4 Immunofluorescence and Immunohistochemical analysis

Renal tissue immunofluorescence and immunohistochemical analyses were conducted using 2 μm paraffin-embedded sections. Following deparaffinization and hydration, the sections underwent antigen retrieval in citrate buffer at high temperature and were subsequently blocked with sheep serum (ZLI-9022, ZSGB-BIO). The sections were then incubated overnight at 4 °C with either a primary anti-mouse IgG antibody (1:200, GB23301, Servicebio), a primary anti-myeloperoxidase (MPO) antibody (1:200, AB_396309, BD PharMingen), anti-neutrophil elastase (NE) antibody (1:200, 27642-1-AP, Proteintech), or an anti-citrullinated histone H3 (CitH3) antibody (1:200, ab5103, Abcam). For immunofluorescence staining, the sections were further incubated with secondary antibodies at room temperature for 1 hour, followed by DAPI (Sigma-Aldrich, St. Louis, MO) nuclear counterstaining and observation under a fluorescence microscope. For immunohistochemical analysis, the sections were stained with 3,3’-diaminobenzidine (DAB) after secondary antibody incubation, and images were captured from five randomly selected positive fields. The positive area of IgG deposition was quantified using ImageJ software (National Institutes of Health), and semi-quantitative scoring was performed using a repeated measures method.

Neutrophils were isolated from the blood of lupus nephritis patients and healthy controls using Ficoll density gradient centrifugation. The isolated neutrophils were subsequently treated with phorbol 12-myristate 13-acetate (PMA, 25 ng/mL, P1585, Sigma-Aldrich), deoxyribonuclease I (DNase I, 1 mg/mL, 11284932001, Roche Diagnostics), and anti-myeloperoxidase (MPO, 1:200, AB_396309, Roche Diagnostics). Immunofluorescence staining was performed using either BD PharMingen antibodies or anti-citrullinated histone H3 (CitH3, 1:200, ab5103, Abcam), as previously described in the literature (23).

### 2.5 Western blot analysis

Western blotting was performed as previously described (AJP and Frontiers). Whole-cell and tissue lysates were prepared using RIPA buffer (Beyotime Biotechnology, Shanghai, China). The following antibodies were utilized: NE (1:1000, 27642-1-AP, Proteintech); Cit H3 (1:1000, ab5103, Abcam); TLR4 (1:1000, 19811-1-AP, Proteintech); MYD88 (1:1000, 67969-1-Ig, Proteintech); IL6 (1:1000, GB11117-100, Servicebio); INOS (1:1000, 18985-1-AP, Proteintech); GAPDH (1:3000, HRP-60004, Proteintech) served as the loading control. The original image files for the western blot can be found in **Supplementary Data Sheet 1**.

### 2.6 RNA-seq processing and analysis

RNA sequencing (RNA-seq) was conducted by Shanghai Ouyi Biomedical Technology Co., Ltd. Initially, total RNA was extracted from kidney samples. Subsequently, the purity and concentration of RNA were assessed, followed by measurement of RNA integrity using an Agilent Bioanalyzer or equivalent method. Samples that met quality control criteria were utilized for library preparation. Transcriptome sequencing was performed on the Illumina HiSeq X Ten platform provided by Novogene Bioinformatics Technology. HTSeq software (version 0.6.0) was employed to align 100 bp paired-end reads to reference genes. Gene expression levels were quantified as fragments per kilobase of transcript per million mapped reads (FPKM). The resulting sequencing data have been deposited in the NCBI Sequence Read Archive (SRA) under accession number PRJNA648341.

Principal Component Analysis (PCA) was employed to reduce the dimensionality of the data and analyze biological reproducibility within groups as well as differences between groups. The dataset was normalized using DESeq2 software, followed by log2 |fold change (FC)| transformation of all gene expression data. A gene was identified as a differentially expressed gene (DEG) if its corrected q-value was less than 0.05 and log2 |FC| exceeded 2. Reactome pathway enrichment analysis and Gene Ontology (GO) enrichment analysis were conducted on the identified DEGs using various databases. Additionally, the TIMER algorithm, which serves as a computational tool for estimating the abundance of diverse immune cell types in complex tissues such as large solid tumors, was utilized in this study to estimate the proportions of immune cell subsets in the kidneys of MRL/lpr mice.

### 2.7 Flow cytometry

After euthanizing the mice, their spleens were harvested, dissociated, and filtered through a 70 μm cell strainer to generate a single-cell suspension. Lymphocytes were subsequently isolated by density gradient centrifugation using lymphocyte separation solution. The following antibodies were utilized for staining: anti-CD3 antibody (E-AB-F1013Q), anti-CD4 antibody (E-AB-F1097S), anti-CD8 antibody (E-AB-F1104J), anti-CD19 antibody (E-AB-F0986E), anti-CD25 antibody (E-AB-F1102D), and anti-Foxp3 antibody (E-AB-F1238E), all sourced from Elabscience (China). Cells were incubated with these antibodies at room temperature in the dark for 30 minutes. The proportions of lymphocyte subsets were then analyzed by flow cytometry using a BD FACS Melody instrument. Flow cytometry data were processed and analyzed using FlowJo software (Tree Star).

### 2.8 RNA isolation and RT-PCR

Total RNA was extracted from mouse kidney, spleen, or neutrophils using the RNA extraction kit (Promega). The concentration and purity of the RNA were assessed using a spectrophotometer. Subsequently, RNA was reverse transcribed into cDNA using HiScript II Q RT SuperMix (Vazyme) in accordance with the manufacturer’s protocol. Quantitative PCR was performed on a Light Cycler 480 II (Roche) using ChamQ SYBR qPCR Master Mix (Vazyme), following the manufacturer’s instructions. The thermal cycling protocol included an initial denaturation step at 95°C for 30 seconds, followed by 40 cycles consisting of denaturation at 95°C for 10 seconds, annealing at 60°C for 30 seconds, and extension at 72°C for 30 seconds. Relative gene expression levels were determined using the 2^-△△Ct^ method, with β-Actin or GAPDH mRNA serving as internal references. All reactions were conducted in triplicate, and the specificity of the amplification products was confirmed by melting curve analysis. The sequence of the primers used in the RT-PCR is shown in **Supplementary Table S1**.

### 2.9 Statistical analysis

Statistical analyses were conducted using GraphPad Prism version 6.0, and all summary data are expressed as the mean ± SD of each independent experiment. Comparisons between groups were performed using a t-test for two-group comparisons or one-way ANOVA for multiple-group comparisons. A *P* value of less than 0.05 was considered to indicate statistical significance.

## 3 Result

### 3.1 DNase I attenuates lupus erythematosus-like symptoms and mitigates renal pathological damage in MRL/lpr mice

**Figure 1:**
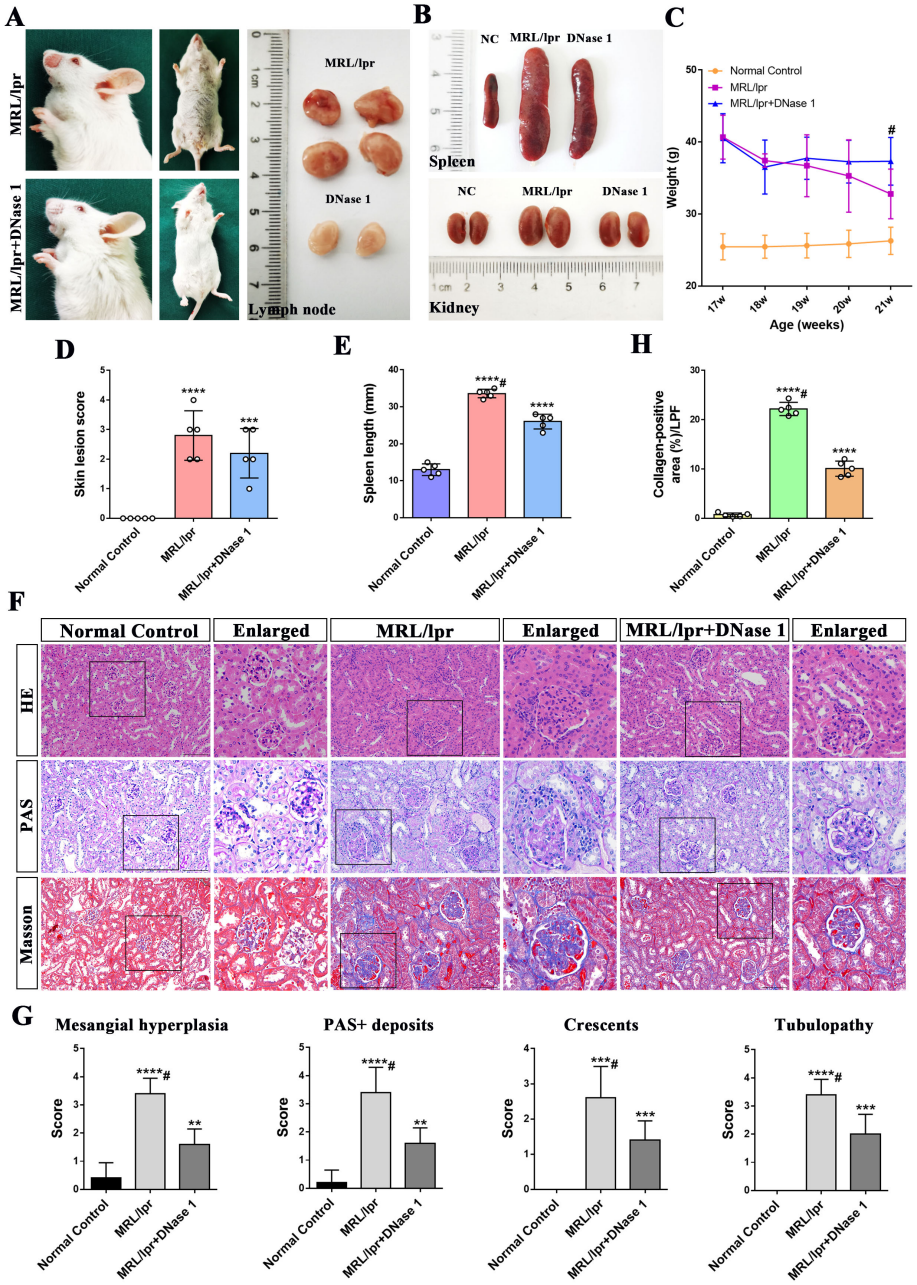
DNase I ameliorates lupus erythematosus-like symptoms and attenuates renal pathological damage in MRL/lpr mice. **(A)** Representative histological images of the skin, lymph nodes, from MRL/lpr and DNase I-treated mice (21 weeks of age). **(B)** Representative histological images of spleen, and kidney from Normal Control (NC), MRL/lpr and DNase I-treated MRL/lpr mice (21 weeks of age). **(C)** Statistical plot of body weight changes in the NC, MRL/lpr and DNase I-treated MRL/lpr group at the beginning and end of the experimental period. **(D)** Assessment of skin lesion scores and **(E)** spleen length in NC, MRL/lpr and DNase I treatment MRL/lpr group. **(F)** Representative HE, PAS, and Masson-stained renal tissue sections from 21-week-old NC, MRL/lpr and DNase I-treated MRL/lpr mice (scale bar, 100 μm). **(G)** Semi-quantitative renal pathological scoring, including mesangial cell proliferation, PAS-positive deposition, tubular injury, and crescentic lesion formation, in the NC, MRL/lpr and the DNase I-treated MRL/lpr group. **(H)** Quantitative analysis of collagen-positive blue staining area (low-power field, LPF) in renal tissues of different experimental group. Data were expressed as means ± SD for groups of five mice. *P < 0.05, **P < 0.01, ***P < 0.001, and ****P < 0.0001 vs. normal control (*t* test). #*P* < 0.05 vs. MRL/lpr mice (Bonferroni correction; two comparisons were made). 17, 18, 19, 20, 21W: 17, 18, 19, 20, 21 weeks of MRL/lpr mice. HE, hematoxylin and eosin; PAS, periodic acid-Schiff.

MRL/lpr mice began to develop skin lesions on the head, face, and trunk at 18 weeks of age. Meanwhile, the lymph nodes, spleen, and kidneys of MRL/lpr mice were markedly enlarged compared to those of normal control (NC) C57BL/6 mice; this finding is consistent with the established phenotype of systemic lupus erythematosus in this model. Lymph nodes from control mice were not included in the figures due to their small size and limited visibility. Following 4 weeks of DNase I treatment, skin lesions and alopecia, as well as lymphadenopathy (**Figures 1A, D**), splenomegaly and renal enlargement (**Figures 1B, E**), were ameliorated. Additionally, weight loss was halted in DNase I-treated mice compared with untreated MRL/lpr mice (**Figure 1C**).

In comparison to normal controls, HE and PAS staining revealed glomerular immune complex deposition, renal interstitial inflammatory cell infiltration, and glomerular crescent formation in the MRL/lpr mice model group at 21 weeks of age. In contrast, the DNase I-treated group exhibited less severe glomerular damage, with only moderate mesangial and endothelial cell proliferation and reduced inflammatory cell infiltration (**Figure 1F**). Masson’s trichrome staining and statistical analysis demonstrated that collagen deposition in the kidneys of MRL/lpr mice was significantly increased compared with NC mice, while collagen deposition was significantly improved after DNase I treatment (**Figures 1F, H**). Additionally, pathological scoring revealed that, compared with the normal control group, the MRL/lpr mouse model group exhibited marked mesangial cell proliferation, PAS-positive deposition, crescentic lesions, and renal tubular injury. However, these indicators of kidney injury were significantly reduced in the DNase I treatment group (**Figure 1G**). Collectively, these findings suggest that lupus-like symptoms were markedly improved and renal pathological damage was attenuated in the LN mouse model after DNase I treatment.

### 3.2 DNase I improved disease activity and renal function while inhibiting the formation of NETs in the kidney of MRL/lpr mice

**Figure 2:**
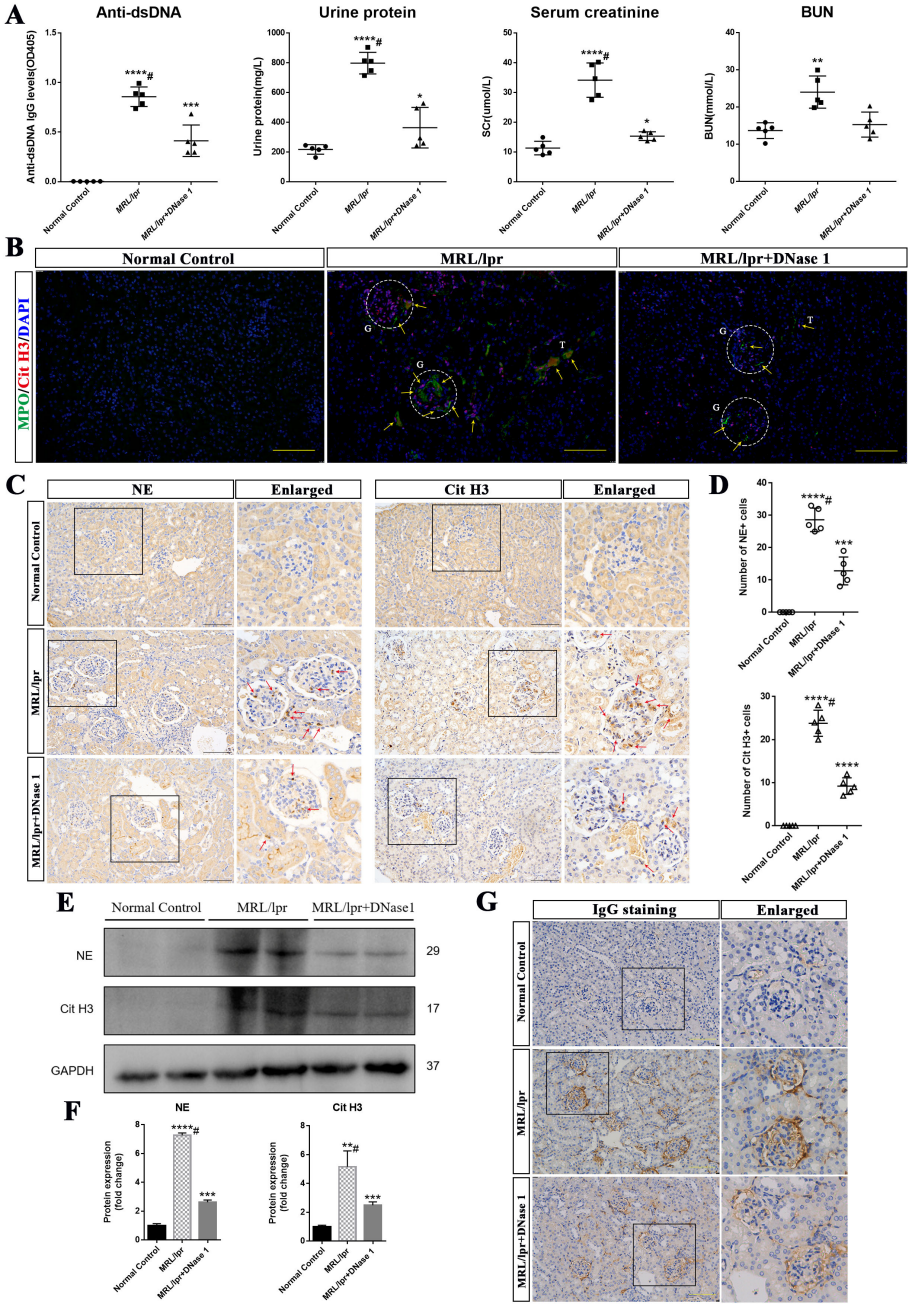
DNase I ameliorated renal function and inhibited the formation of neutrophil extracellular traps (NETs) in MRL/lpr mice. **(A)** Levels of anti-double-stranded DNA antibodies (Anti-dsDNA), urinary protein, serum creatinine (SCr), and blood urea nitrogen (BUN) in Normal Control (NC), MRL/lpr and DNase I-treated MRL/lpr mice at 21 weeks of age (n=5). **(B)** Representative immunofluorescence images (scale bar, 100 μm) showing renal MPO (green), Cit H3 (red), and DAPI (blue) staining in NC, MRL/lpr and DNase I-treated MRL/lpr group. Glomeruli was located with capital G and white circle. Capital T indicate the renal tubules. Yellow arrows indicate MPO and Cit H3 double-positive NETs. **(C)** Immunohistochemistry analysis of NE and Cit H3 from the NC, MRL/lpr and DNase I treatment MRL/lpr group at 21 weeks of age. Red arrows indicate NE or Cit H3 positive cells. (scale bar, 100 μm) **(D)** Scatter plot statistical analysis of the number of NE and Cit H3 positive cells in different group of kidney sections. **(E, F)** Western blot analysis was performed to assess the expression levels of NE and Cit H3 in kidney tissues from the NC, MRL/lpr and DNase I-treated MRL/lpr group. **(G)** Representative images depicting IgG deposition in the kidneys of NC, MRL/lpr, and DNase I-treated MRL/lpr mice (scale bar, 100 μm). Data were expressed as means ± SD for groups of five mice. *P < 0.05, **P < 0.01, ***P < 0.001, and ****P < 0.0001 vs. normal control (*t* test). #*P* < 0.05 vs. MRL/lpr mice (Bonferroni correction; two comparisons were made).

To further investigate whether DNase I exerts an effect on disease activity and renal function recovery in LN mice, we measured LN autoantibodies and molecular markers associated with renal function. At 21 weeks of age, serum concentrations of anti-dsDNA, ANA, creatinine, urea nitrogen, and urinary protein were significantly elevated in the MRL/lpr mice model group but markedly reduced in the DNase I-treated group compared with the normal control group. Notably, serum urea nitrogen levels returned to normal in the DNase I-treated group (**Figure 2A**). Immunohistochemical staining revealed a reduction in IgG antibody deposition in the kidneys of LN mice following DNase I treatment (**Figure 2G**), suggesting that DNase I reduced the deposition of antibody immune complexes (ICs) in the kidneys of MRL/lpr mice.

Among the various NETs-related active components, MPO, neutrophil elastase (NE) and CitH3 detection are widely utilized for the identification of NETs across different diseases (24, 25). Given prior studies demonstrating the crucial role of NETs in LN pathogenesis (23), we also examined MPO and CitH3 expression in the kidneys of different groups of mice using immunofluorescence. Our results demonstrated that MPO and CitH3 exhibited strong positive expression in the kidneys of MRL/lpr mice compared to those of normal control mice, and the NETs were predominantly deposited in the glomerulus, with only a portion of deposition observed in the renal interstitium. Notably, the expression levels of these markers were markedly reduced following DNase I treatment (**Figure 2B**). Furthermore, immunohistochemical analysis revealed that the number of NE- and CitH3-expressing cells in the kidneys of MRL/lpr mice was significantly higher compared to NC mice, which exhibited minimal expression. Notably, following DNase I treatment, a marked reduction in NE- and CitH3-positive cells was observed (**Figures 2C, D**). Western blot analysis further confirmed that the renal expression of NE and CitH3 was significantly decreased in DNase I-treated MRL/lpr mice compared to untreated MRL/lpr mice (**Figures 2E, F**). These results suggest that DNase I can substantially attenuate disease activity and renal dysfunction in a mouse model of LN, inhibit the activation of NETs in the kidney, which is closely associated with the pathogenesis of the disease, and thereby mitigate renal damage.

### 3.3 RNA-seq revealed the activation of neutrophils in MRL/lpr mice kidneys, and downregulation of immune cell chemotaxis and upregulation of damage repair pathway following DNase I treatment

**Figure 3:**
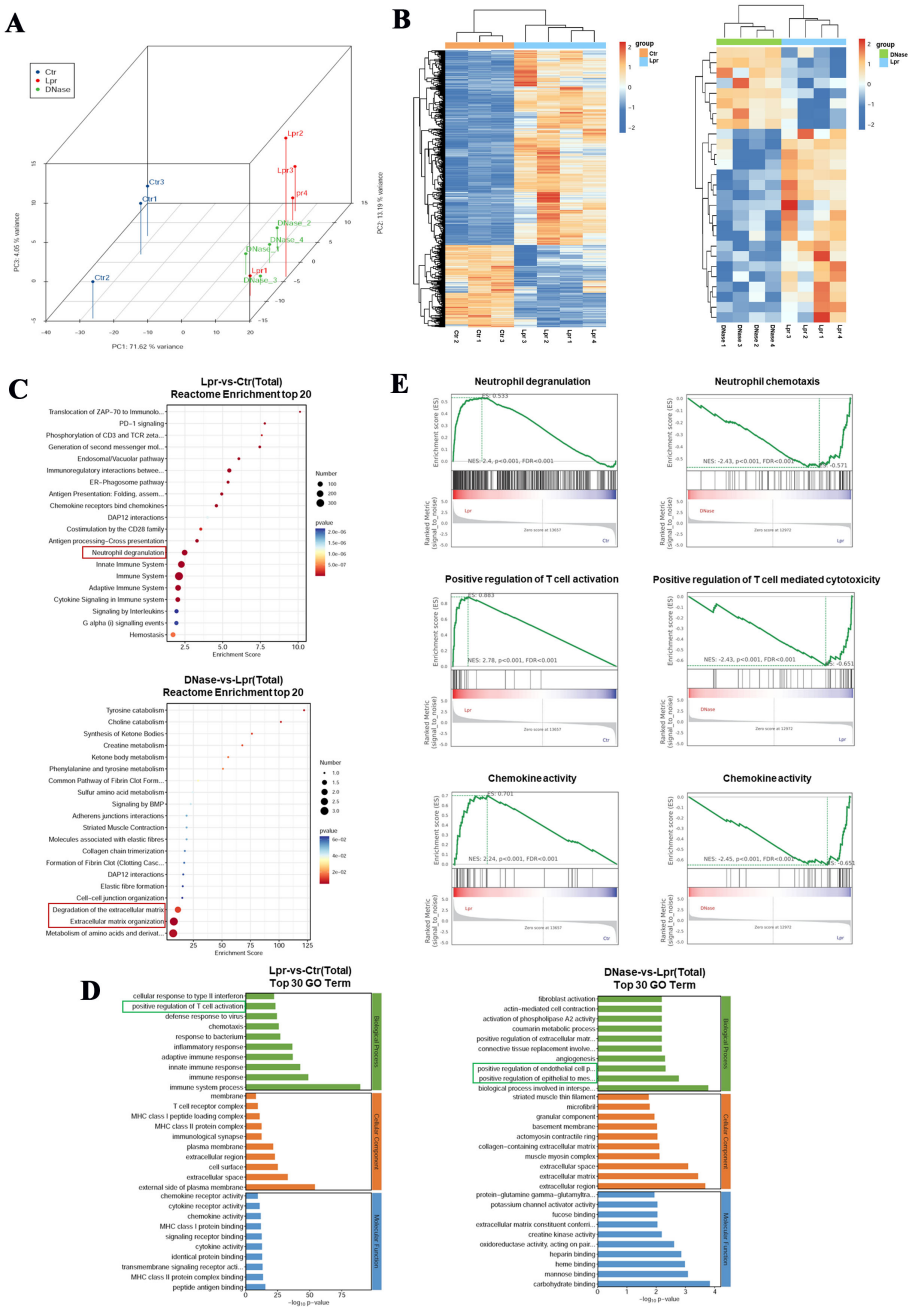
Neutrophils are activated in the kidneys of MRL/lpr mice, and DNase I can inhibit the activation and chemotaxis of immune cells. **(A)** Principal component analysis (PCA) of RNA-seq data from the kidneys of normal control (Ctr), MRL/lpr (Lpr), and DNase I-treated MRL/lpr (DNase) mice at 21 weeks of age. **(B)** Cluster heatmaps of differentially expressed genes between the normal control and the MRL/lpr group, as well as between the DNase I treatment MRL/lpr and the MRL/lpr group. Reactome enrichment analysis **(C)** and Gene Ontology (GO) enrichment analysis **(D)** of differentially expressed genes. **(E)** Gene-set enrichment analysis (GSEA) was used to analyze potential functional enrichments of differentially expressed genes in the kidneys of mice between different groups.

To further investigate the potential molecular mechanisms underlying DNase I-targeted NETs in the treatment of LN in a mouse model, we conducted RNA sequencing on kidney tissues from four 21-week-old MRL/lpr mice (Lpr), four MRL/lpr mice treated with DNase I (DNase), and three sex- and age-matched healthy control mice (Ctr). We identified 1265 upregulated differentially expressed genes (DEGs) and 533 downregulated DEGs in MRL/lpr mice kidneys compared to controls. In contrast, the DNase I treatment group showed 8 upregulated genes and 19 downregulated genes compared to the MRL/lpr mice group. Hierarchical clustering analysis of DEGs revealed distinct gene expression profiles across the three groups. Additionally, principal component analysis (PCA) demonstrated that the three groups exhibited both high biological reproducibility and inter-sample differences (**Figures 3A, B**). Functional enrichment analysis using Reactome enrichment analysis indicated that, in addition to being enriched in pathways associated with innate/adaptive immune system activation, the kidney-specific DEGs in the LN mouse model were also significantly enriched in neutrophil activation pathways. Conversely, in the DNase I treatment group, more differentially expressed genes were enriched in pathways associated with metabolism of amino acid and derivatives, extracellular matrix organization, and degradation of the extracellular matrix.

Gene Ontology (GO) enrichment analysis further revealed that DEGs in MRL/lpr mice kidneys were predominantly enriched in immune system activation, inflammatory response, and T cell activation pathways, whereas DEGs in the DNase I treatment group were primarily enriched in pathways of EMT positive regulation and epithelial cell proliferation related to damage repair (**Figures 3C, D**). Gene-set enrichment analysis (GSEA) revealed that the responses associated with neutrophil degranulation, T cell activation, and chemokine activity were significantly upregulated in MRL/lpr mice kidneys. Following DNase I treatment, neutrophil chemotaxis, T cell-mediated cytotoxicity, and chemokine-related molecular pathways were significantly downregulated (**Figure 3E**). Collectively, these findings indicate that immune cell-related signaling pathways, particularly those involving neutrophils and T cells, were activated in MRL/lpr mice kidneys. Conversely, DNase I treatment inhibited immune cell activation and chemotaxis pathways while promoting renal injury repair pathways.

### 3.4 DNase I attenuated the infiltration of cytotoxic immune cells and down-regulated the expression of molecules associated with inflammatory damage pathway and NETs formation

**Figure 4:**
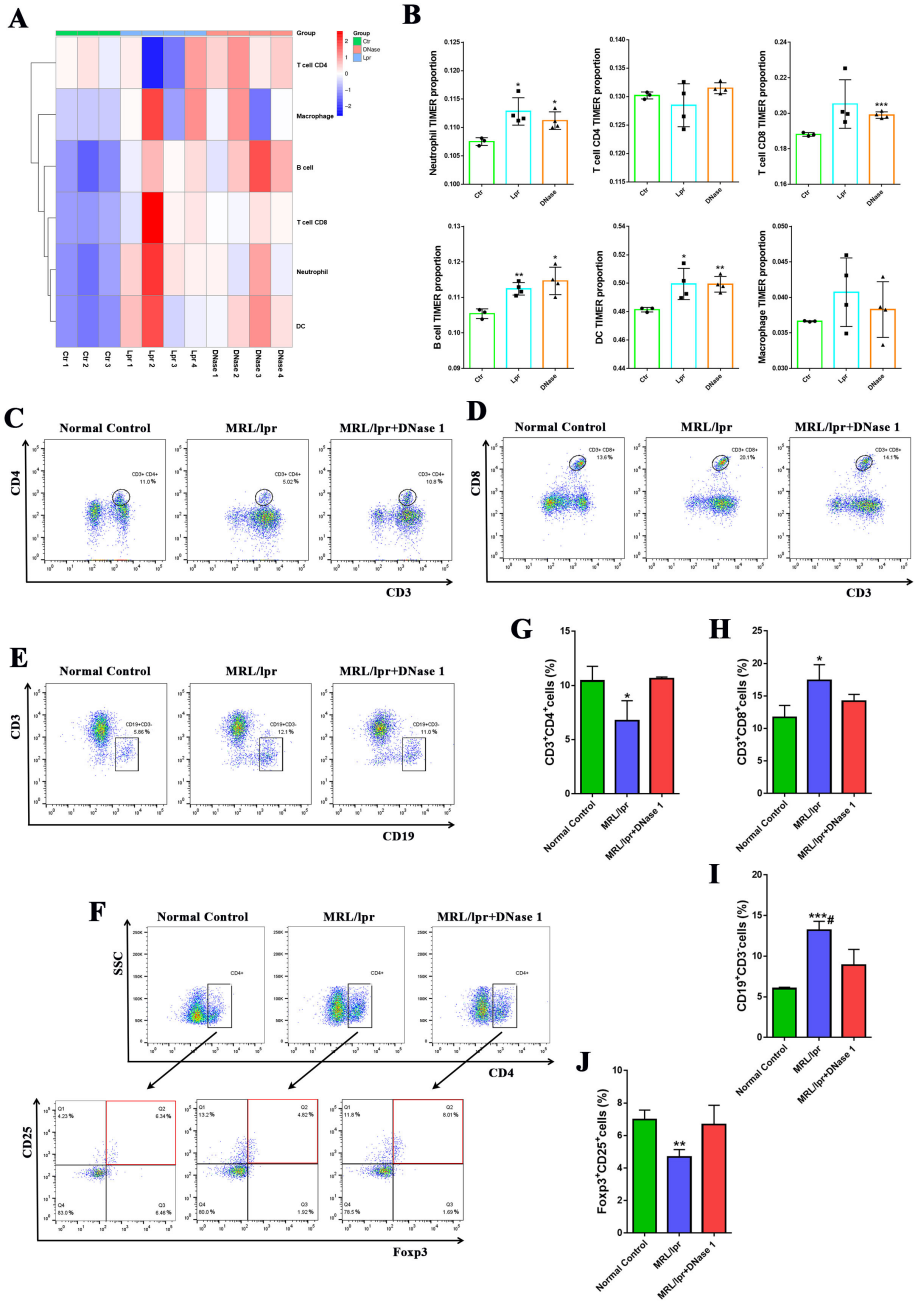
DNase I treatment decreased the number of immune inflammatory cells in the kidneys and spleens of mice in the MRL/lpr model. **(A, B)** The TIMER algorithm was used to assess the proportion of immune cell infiltration in the kidneys of control (Ctr), MRL/lpr (Lpr), and DNase I-treated MRL/lpr (DNase) mice at 21 weeks of age. The percentages of CD3+CD4+ T cell subsets **(C, G)**, CD3+CD8+ T cell subsets **(D, H)**, and CD3-CD19+B cell subsets **(E, I)** in the spleen were quantified using flow cytometry across the normal control, MRL/lpr, and DNase I treatment MRL/lpr group. **(F, J)** Flow cytometric and quantitative analysis of CD4+CD25+Foxp3+ regulatory T cell (Treg) percentages in the spleens of mice from each experimental group were also conducted. Data were expressed as means ± SD for groups of three mice. *P < 0.05, **P < 0.01, and ***P < 0.001 vs. normal control (*t* test). #*P* < 0.05 vs. MRL/lpr mice (Bonferroni correction; two comparisons were made).

Our previous studies have demonstrated that immune cell activation and infiltration are critical in mediating renal inflammatory injury in lupus nephritis (23). RNA-seq analyses further indicated that differentially expressed genes in LN kidneys were predominantly enriched in immune cell-mediated inflammation pathways. To investigate the impact of DNase I on immune cell infiltration in the LN mouse model kidney, we employed the TIMER algorithm to estimate the proportions of various immune cell types within the kidney. The results indicated that the numbers of neutrophils, cytotoxic CD8+ T cells, B cells, dendritic cells (DCs), and macrophages were significantly increased in the kidneys of the MRL/lpr mice group compared to the normal control group (Ctr). Conversely, the number of CD4+ T cells was reduced. In the DNase I treatment group, however, the counts of these immune cells were decreased compared with the MRL/lpr mice group, while the number of CD4+ T cells was increased (**Figures 4A, B**). Flow cytometric analysis using CD3, CD4, and CD8 as markers for T cell identification, we observed that the proportion of CD3+CD4+ T cells in the spleen of the MRL/lpr mice group was significantly lower than that of the normal control group, whereas the proportion of CD3+CD8+ T cells was significantly higher. Following DNase I treatment, the proportions of both CD3+CD4+ and CD3+CD8+ T cells in the spleen reverted tended to the normal control group (**Figures 4C, G, D, H**). Additionally, the proportion of CD3-CD19+ B cells in the spleen was significantly increased in the MRL/lpr mice group but significantly decreased following DNase I treatment (**Figures 4E, I**). Moreover, using CD4, CD25, and Foxp3 as markers for regulatory T cells (Tregs), we found that Treg cells, which function as negative immune regulators, were significantly reduced in the spleen of LN model, while DNase I treatment effectively restored their numbers (**Figures 4F, J**).

**Figure 5:**
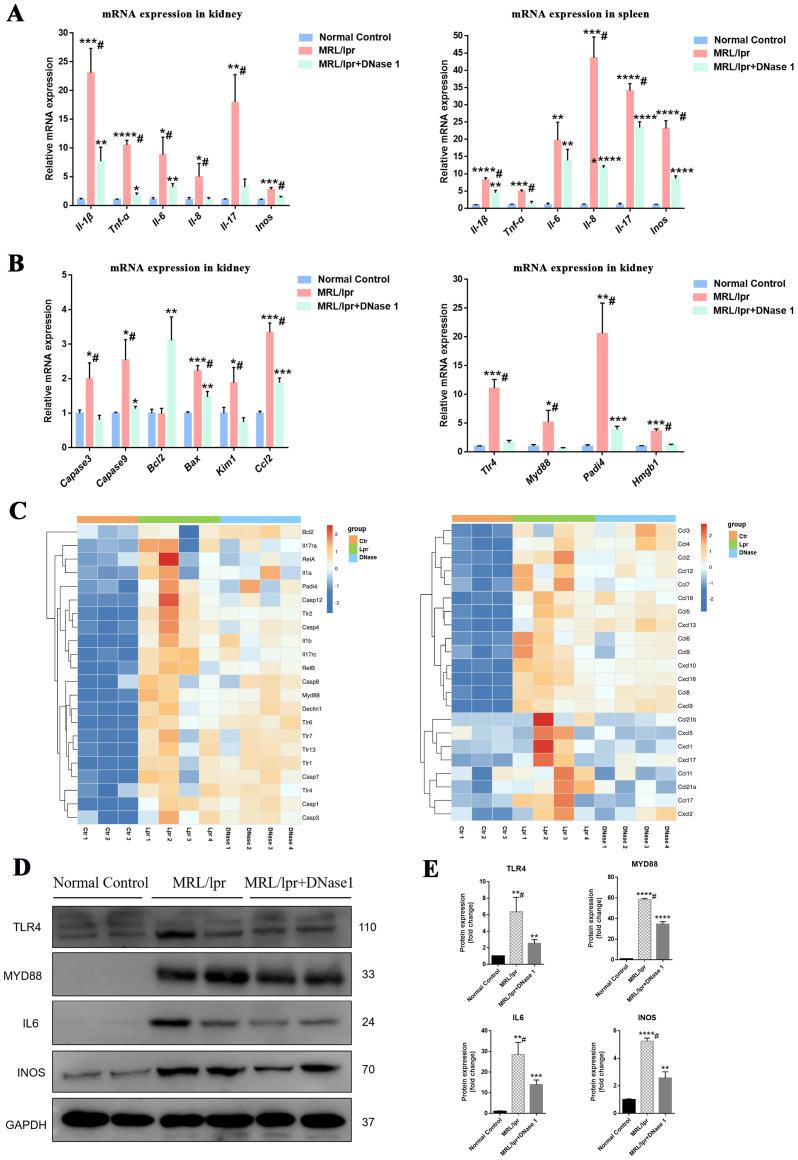
DNase I downregulates the expression of inflammatory damage molecules and NETs-related molecules in the MRL/lpr model. **(A)** qRT-PCR was performed to assess the mRNA expression levels of inflammatory molecules in the kidneys and spleens of normal control, MRL/lpr, and DNase I-treated MRL/lpr mice at 21 weeks of age. **(B)** qRT-PCR was conducted to evaluate the mRNA expression levels of apoptotic molecules, chemokines, and genes associated with NETs formation in kidney tissues from the normal control, MRL/lpr, and DNase I treatment MRL/lpr group. **(C)** RNA-seq data illustrating the expression profiles of inflammatory pathway molecules, damage apoptosis molecules, NETs-associated molecules, and chemokines in the kidneys of normal control (Ctr), MRL/lpr (Lpr), and DNase I-treated MRL/lpr (DNase) mice. **(D, E)** Western blotting was employed to detect and quantify the protein expression levels of TLR4, MYD88, IL6, and INOS in the kidneys of 21-week-old mice from the normal control, MRL/lpr, and DNase I treatment MRL/lpr group. Data were expressed as means ± SD for groups of three mice. *P < 0.05, **P < 0.01, ***P < 0.001, and ****P < 0.0001 vs. normal control (*t* test). #*P* < 0.05 vs. MRL/lpr mice (Bonferroni correction; two comparisons were made).

The infiltration and activation of immune cells frequently result in inflammatory damage to tissues and organs. DNase I may suppress the expression of inflammatory molecules by regulating the activation of immune cells in LN, thereby mitigating tissue and cellular damage. Real-time PCR analysis revealed that the expression levels of inflammatory factors (IL-1β, TNF-α, IL-6, IL-8, IL-17, and INOS) in the kidneys and spleens of MRL/lpr mice were significantly upregulated compared to those in control mice. However, following DNase I treatment, the expression of these inflammatory genes was significantly downregulated (**Figure 5A**). Additionally, the mRNA expression levels of apoptosis and injury genes (Caspase3, Caspase9, Bax, and Kim1) and the chemokine CCL2 were increased in the MRL/lpr mice group but decreased in the DNase I-treated group. Conversely, the anti-apoptotic molecule Bcl2 was upregulated in the DNase I-treated group. Consistent with the expression of inflammatory factors, molecules involved in inflammatory cell signaling pathways (TLR4, MYD88, and HMGB1), as well as the key molecule for NETs formation, PADI4, were highly expressed in the LN model but significantly downregulated in the DNase I-treated group (**Figure 5B**). RNA-seq gene cluster analysis of the kidneys confirmed that the expression patterns of inflammatory factors, signaling pathway molecules, damage- and apoptosis-related molecules, and chemokines across different groups aligned with the findings from RT-PCR (**Figure 5C**). Western blot analysis further validated that the protein levels of pathway molecules TLR4 and MYD88, as well as inflammatory molecules IL6 and INOS, were significantly upregulated in the MRL/lpr mice group, whereas their expression was significantly reduced in the DNase I-treated group (**Figures 5D, E**). Collectively, our results indicate that DNase I could inhibit the expression of inflammatory injury pathway molecules by reducing immune cell infiltration in the kidneys, and simultaneously down-regulate the expression of key NETs-formation molecules and chemokines *in vivo*.

### 3.5 DNase I inhibited NETs formation, regulated inflammatory pathways and the expression of key molecules of NETs *in vitro*

**Figure 6:**
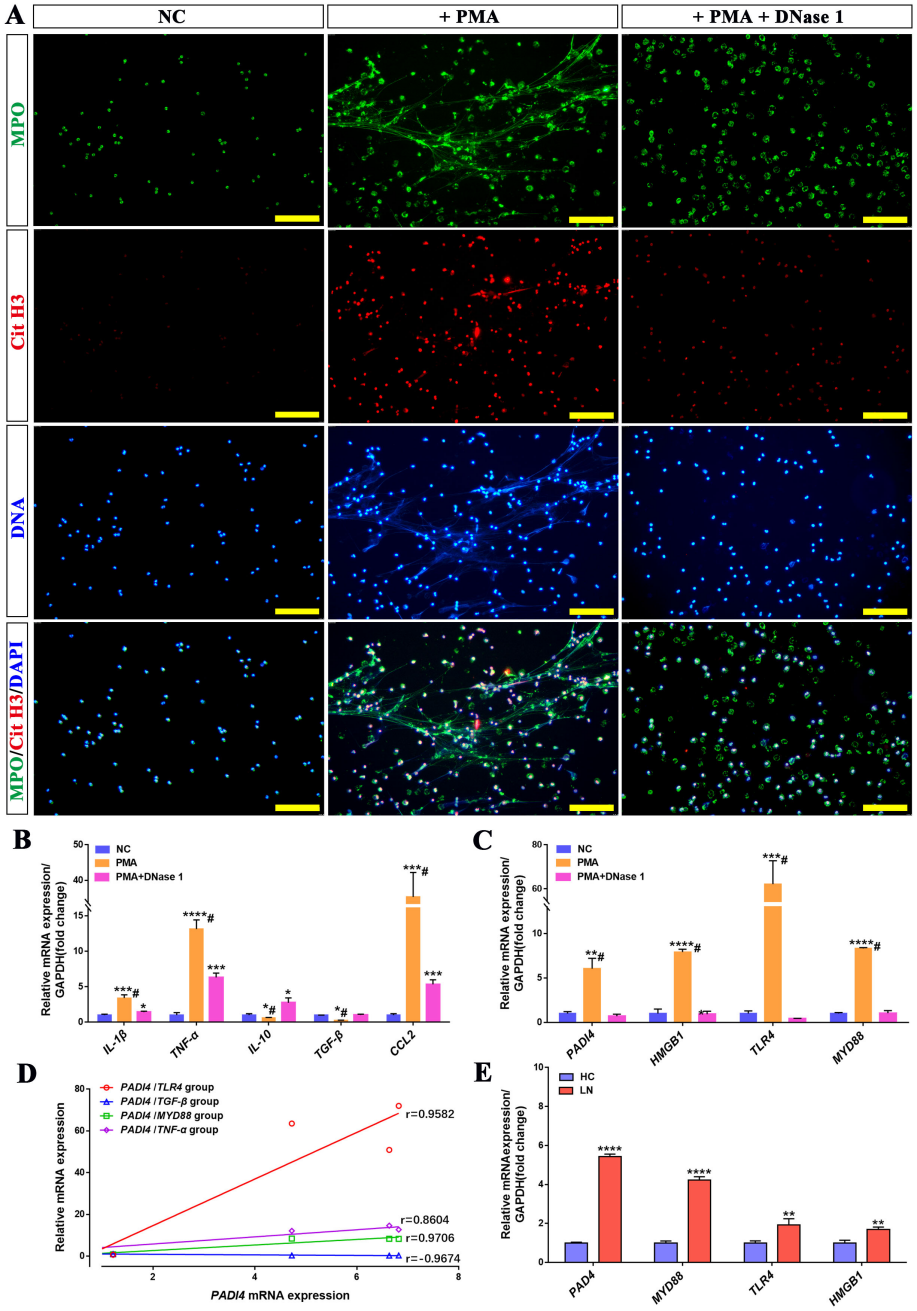
DNase I can reduce NETs formed by activated neutrophils *in vitro* and inhibit the expression of inflammatory molecules and NETs-related genes. **(A)** Immunofluorescence analysis of NETs (MPO in green, Cit-H3 in red, DAPI in blue; scale bar, 100 μm) in PMA-treated neutrophils, PMA + DNase I-treated neutrophils, or normal control (NC). **(B)** qRT-PCR was used to quantify the mRNA expression levels of IL-1β, TNF-α, IL-10, TGF-β, and CCL2 in human neutrophils from the NC, PMA-treated, and PMA + DNase I-treated group. **(C)** The mRNA expression levels of NETs-related molecules, including PADI4, HMGB1, TLR4, and MYD88, were analyzed across the NC, PMA-treated, and PMA + DNase I-treated group. **(D)** The correlation between PADI4 mRNA expression and TLR4, TGF-β, MYD88, TNF-α mRNA expression in neutrophils of different groups (TLR4, p<0.0001; TGF-β, p<0.0001; MYD88, p<0.0001; TNF-α, p=0.0029). **(E)** qRT-PCR was conducted to assess the mRNA expression levels of key NETs-associated molecules—PADI4, HMGB1, TLR4, and MYD88—in peripheral blood neutrophils isolated from healthy controls (HC) and patients with lupus nephritis (LN). Data were expressed as means ± SD for three neutrophil samples. *P < 0.05, **P < 0.01, ***P < 0.001, and ****P < 0.0001 vs. NC or HC (*t* test). #*P* < 0.05 vs. PMA-treated group (Bonferroni correction; two comparisons were made).

Based on the *in vivo* results, DNase I can attenuate the immune response and alleviate renal inflammatory injury. To further investigate whether DNase I inhibits the expression of inflammatory genes and chemokines by targeting the degradation of NETs, we isolated neutrophils from healthy human donors and treated them with phorbol 12-myristate 13-acetate (PMA), a protein kinase C activator, to induce neutrophil activation and NETs formation *in vitro*. Immunofluorescence staining results demonstrated that PMA-treated neutrophils formed a filamentous network structure, with high expression of myeloperoxidase (MPO) and citrullinated histone H3 (CitH3), which serve as markers of NETs formation. Notably, upon addition of DNase I, the expression levels of MPO and CitH3 were significantly reduced, and the filamentous network structure was markedly diminished (**Figure 6A**).

Compared with the normal control neutrophil group (NC), the expressions of pro-inflammatory genes IL-1β, TNF-α, and chemokine CCL2 were significantly upregulated in the PMA-treated group, whereas the expressions of anti-inflammatory and repair-associated genes IL-10 and TGF-β were downregulated. In the PMA+DNase I group, the mRNA levels of IL-1β, TNF-α, and CCL2 were reduced relative to the PMA-treated group, although they remained significantly higher than those in the control group; conversely, the mRNA levels of IL-10 and TGF-β were increased. Furthermore, PADI4, a pivotal molecule involved in NETs formation, along with inflammatory pathway molecules (TLR4, MYD88, and HMGB1), exhibited robust expression in the PMA-treated group. Notably, their mRNA levels were substantially attenuated in the PMA+DNase I group (**Figures 6B, C**). Correlation analysis further revealed that the mRNA expression of PADI4, was positively correlated with the expression of inflammatory pathway molecules (MYD88, IL-1β, TNF-α) and negatively correlated with the expression of injury repair gene TGF-β. Similarly, the mRNA expression levels of PADI4 and inflammatory pathway molecules (TLR4, MYD88, and HMGB1) were significantly upregulated in neutrophils isolated from LN patients compared to healthy control (HC) neutrophils (**Figures 6D, E**). These results suggest that neutrophils activate and up-regulate the expression of inflammatory pathway molecules, inflammatory genes and chemotactic molecules, and promote the formation of NETs, while DNase I can inhibit the formation of NETs and reduce the activation of inflammatory pathways and immune cell chemotaxis. In addition, the expression of PADI4, an important molecule in the formation of NETs, was positively correlated with the expression of inflammation-related molecules. These molecules were also significantly activated in neutrophils from LN patients.

### 3.6 The expression of key molecules related to NETs and inflammation in MRL/lpr mice kidneys are progressively up-regulated over time, correlating positively with NETs formation in the kidney

**Figure 7:**
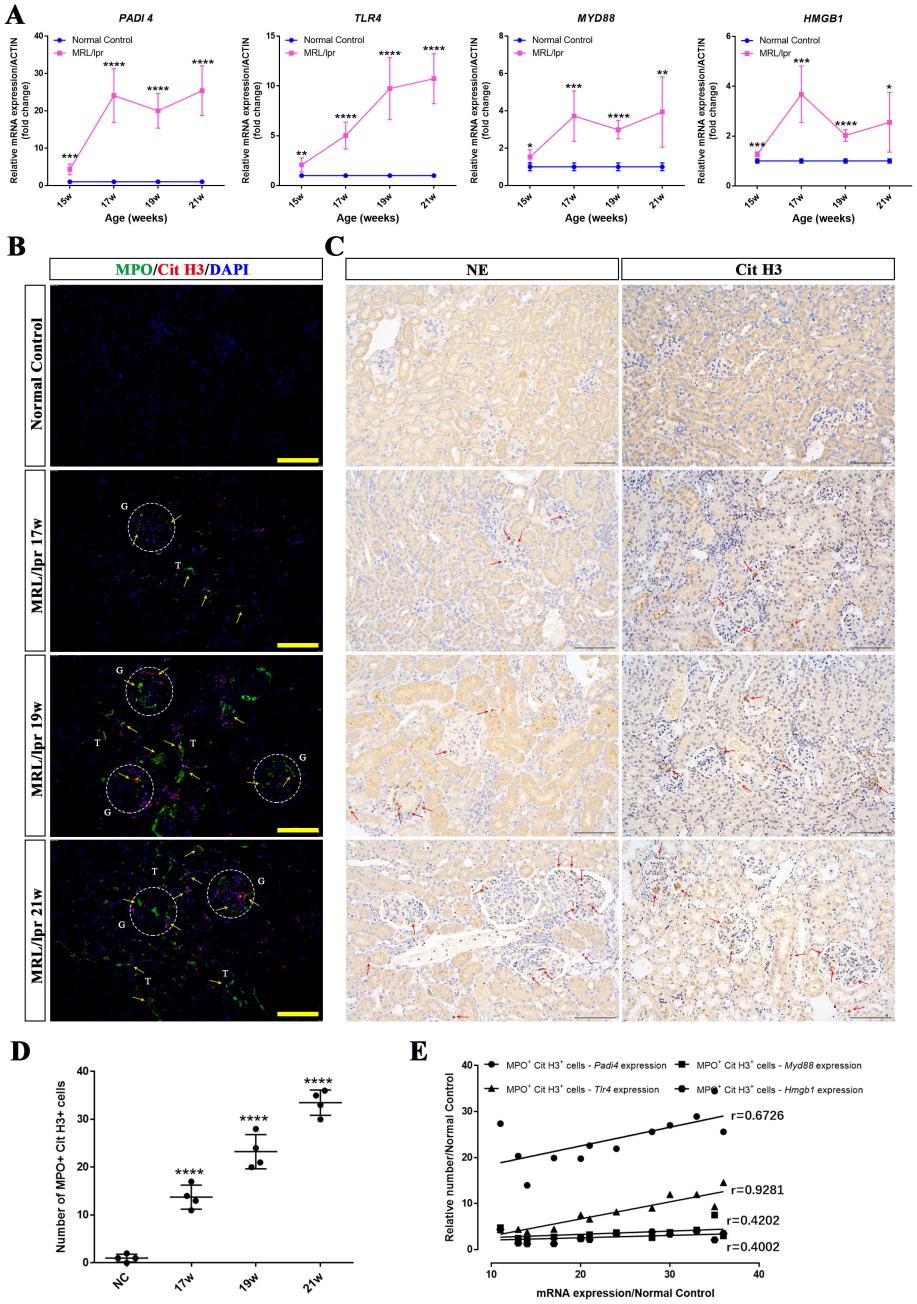
The expression levels of NETs-related molecules were up-regulated in a time-dependent manner and showed a positive correlation with NETs formation in the kidneys of MRL/lpr mice. **(A)** qRT-PCR was performed to measure the mRNA expression of NETs-associated molecules, including PADI4, HMGB1, TLR4, and MYD88, in kidney tissues from normal control (NC) and MRL/lpr mice at different weeks of age (15, 17, 19, and 21 weeks). **(B)** Representative immunofluorescence images of NETs formation in kidney sections are shown (MPO: green; Cit-H3: red; DAPI: blue; scale bar: 100 μm) from both normal control (NC) and MRL/lpr mice at different weeks of age (17, 19, and 21 weeks). Glomeruli was located with capital G and white circle. Capital T indicate the renal tubules. Yellow arrows indicate MPO and Cit H3 double-positive NETs. **(C)** Immunohistochemistry analysis of NE and Cit H3 from the different groups. Red arrows indicate NE or Cit H3 positive cells. (scale bar, 100 μm) **(D)** Scatter plot statistical analysis of the number of MPO and Cit H3 double positive cells in kidney sections of NC and MRL/lpr mice at different weeks of age. **(E)** statistical analysis of the correlation between the number of MPO and Cit H3 double positive cells and the key molecular markers (PADI4, p=0.0165; TLR4, p<0.0001; MYD88, p=0.1738; HMGB1, p=0.1974). Data were expressed as means ± SD for groups of three mice. *P < 0.05, **P < 0.01, ***P < 0.001, and ****P < 0.0001 vs. normal control (*t* test). 15, 17, 19, 21W: 15, 17, 19, 21 weeks of mice.

PADI4, TLR4, MYD88 and HMGB1 are key molecules closely associated with the formation and function of NETs. We used RT-PCR to evaluate the mRNA expression levels of these genes in the kidneys of MRL/lpr mice at different weeks of age (15 weeks, 17 weeks, 19 weeks, and 21 weeks). Compared with age-matched control mice, the expression levels of PADI4, TLR4, MYD88 and HMGB1 were significantly upregulated, and the fold expression of these genes in MRL/lpr group gradually upregulated with the increase of week (**Figure 7A**). Consistently, immunofluorescence staining demonstrated that the number of MPO+ CitH3+ double-positive cells in the kidneys of 17-week, 19-week, and 21-week MRL/lpr mice was significantly higher than that in normal control mice, exhibiting a gradual upward trend (**Figures 7B, D**). Immunohistochemical staining further revealed that the number of NE- and CitH3-expressing cells in the kidneys of MRL/lpr mice at 17, 19, and 21 weeks of age were significantly increased compared to the normal control group (**Figure 7C**). Correlation analysis further revealed that the mRNA expression of PADI4, TLR4, MYD88 and HMGB1 was positively correlated with the number of MPO+ CitH3+ double-positive cells (**Figure 7E**). Collectively, these findings, in conjunction with the inhibitory effect of DNase I on NETs degradation, suggest that NETs formation in MRL/lpr mice promotes the aberrant expression of PADI4, TLR4, MYD88 and HMGB1, which subsequently induces NETs formation, thereby establishing a closed loop of renal inflammatory injury. DNase I can block the inflammatory damage loop mediated by NETs, and these molecules may serve as potential molecular markers for the formation and function of NETs in LN.

## 4 Discussion

**Figure 8:**
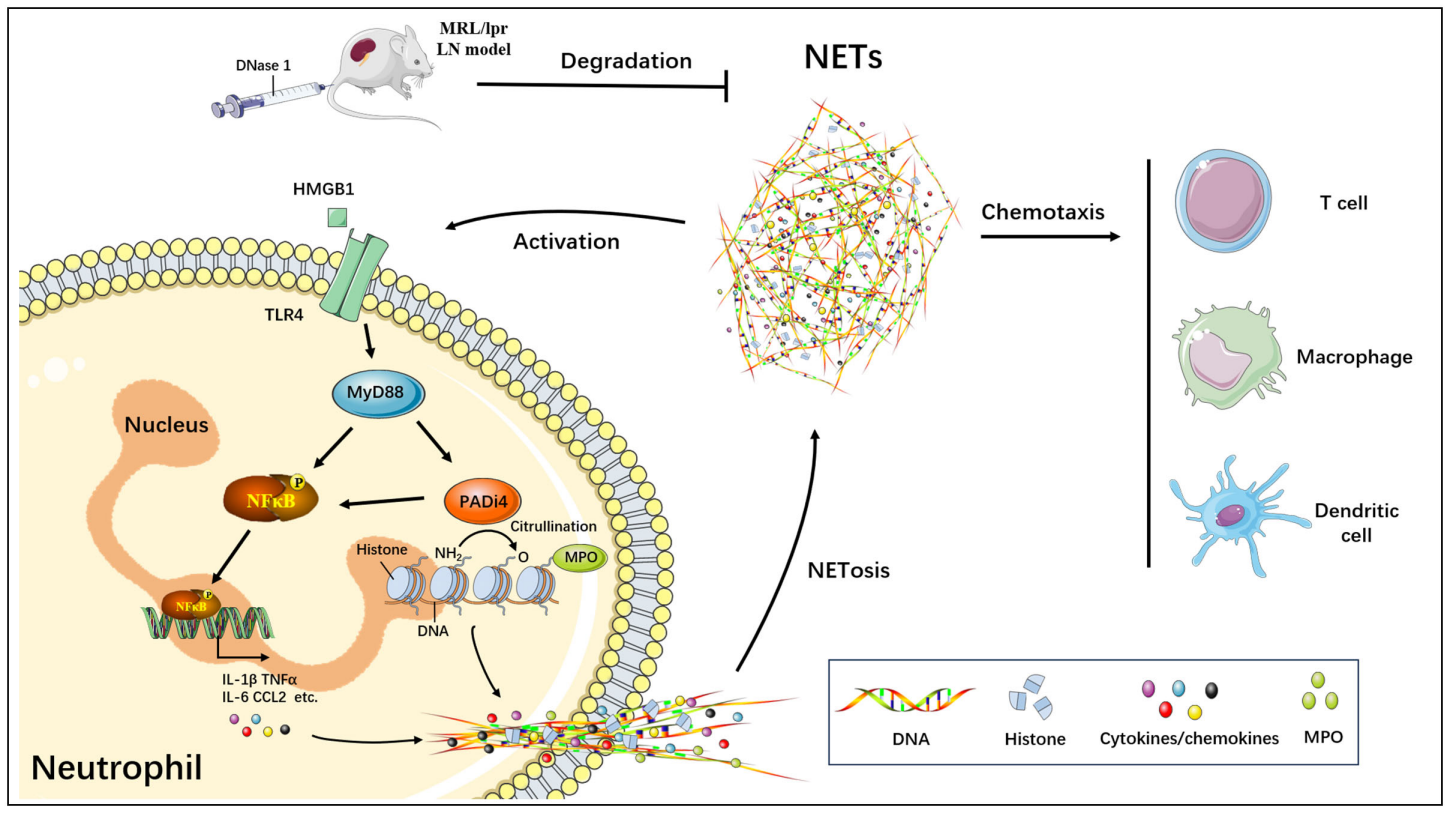
Schematic model of targeted blockade of NETs generation by DNase I and alleviation of NETs-mediated renal inflammatory injury.

Our current study demonstrated that NETs formed through neutrophil activation in the MRL/lpr mice model induced renal inflammatory damage, consistent with prior findings (26, 27). However, the precise regulatory mechanisms underlying NETs formation and function in LN remain to be fully elucidated. In this study, we identified that NETs enhance the expression of PADI4, a critical molecule in NETs formation, by upregulating key inflammatory signaling pathway components such as TLR4, MYD88, NFκB, IL-1β, IL-6, and CCL2 in neutrophils. This process facilitates the accumulation of NETs and promotes the infiltration of immune and inflammatory cells, including T cells and macrophages, thereby contributing to renal inflammatory injury (**Figure 8**). Furthermore, both our research and studies by other investigators have confirmed that targeted degradation of NETs using DNase I significantly alleviates the onset and progression of NETs-mediated renal injury in LN (23, 28). Consequently, the targeted inhibition of NETs can effectively mitigate renal tissue inflammation in LN, representing a promising therapeutic target for the prevention and treatment of this condition.

Although the presence of DNase I inhibitors and anti-NETs antibodies in patients has impeded the broader application of DNase I in humans (6), as a NETs-degrading enzyme, DNase I has been extensively utilized in animal models of NETs-mediated inflammatory tissue and organ damage (21, 22, 29, 30). Recent studies employing single-cell RNA-sequencing technology revealed heterogeneity in neutrophil pancreatic infiltration in an acute pancreatitis mouse model. Subsequent investigations demonstrated that NETs formed through neutrophil activation induced pancreatic inflammatory damage, while DNase I treatment significantly reduced NETs and ameliorated the inflammatory damage of the pancreas (31). Similarly, in a cerebral cavernous hemangioma animal model, RNA-seq analysis indicated that endothelial cells expressed chemokines that recruited neutrophils to form NETs. Following DNase I treatment, vascular inflammatory lesions were markedly ameliorated (32). Our research team conducted RNA sequencing and analysis on the kidneys of MRL/lpr mice. The findings revealed that the infiltration and activation of immune cells, including neutrophils, as well as the expression of inflammatory, injury-related, apoptotic, and chemotactic molecules were significantly upregulated in the kidney tissue. Conversely, following DNase I treatment, there was a marked reduction in the infiltration and activation of immune cells, along with decreased expression of inflammatory and injury-associated molecules. Additionally, an increase in the expression of anti-apoptotic and repair-related molecules was observed. Furthermore, compared to the model group, the pathway associated with extracellular matrix (ECM) degradation and remodeling was significantly activated in the DNase I-treated group. This phenomenon may be attributed to the ability of NETs to directly interact with ECM components, thereby promoting their remodeling and deposition (33, 34). Consequently, the application of DNase I plays an important role in exploring the mechanism of NETs-induced tissue and organ inflammatory damage in animal models, but the specific mechanism needs to be further studied.

It is widely recognized that NETs can induce inflammatory damage in various tissue and organ-damaging diseases via multiple mechanisms, including inflammasome activation (35), TLR7 and TLR9 activation through autoantigen complexes such as LL37-DNA (36), promoting macrophage pyroptosis (37), or processing and activation of the IL-36 cytokine (38, 39). In this study, we observed that the mRNA and protein expressions of key inflammatory pathway molecules, including TLR4, MYD88, and IL-6, were significantly upregulated in the kidneys of the LN mouse model, and were down-regulated with the improvement of renal inflammation after DNase I treatment. This led us to concern that the inflammatory injury of nephritis in LN may be closely related to the activation of TLR4/MYD88 pathway mediated by NETs. Previous studies have shown that highly basic histones, as one of the important components of NETs, can bind to TLR4 and trigger inflammatory response, thereby activating downstream signaling pathways to produce proinflammatory cytokines, chemokines and other inflammatory mediators (40, 41). The pro-inflammatory activity of NETs in inflammatory diseases is critically dependent on the activation of MYD88/NF-κB downstream signaling pathways (20). In a recent study investigating NETs-mediated acute lung injury, researchers demonstrated that high mobility group box 1 (HMGB1), a damage-associated molecular patterns (DAMPs), promotes NETs formation via the activation of the TLR4/MYD88 pathway (42). Our findings from animal models, cellular experiments, and patient samples confirmed that the HMGB1 gene was significantly upregulated. Furthermore, histone proteins and HMGB1 function as DAMPs to enhance the activation of PADI4, a key molecule in NETs formation, through TLR4 and TLR9 signaling pathways in ischemic liver injury, thereby facilitating NETs formation (21). Consequently, NETs generated by neutrophil activation in LN may interact with HMGB1 and other molecules to induce the activation of TLR4/MYD88/NF-κB downstream signaling pathways, activate PADI4, and promote the release of inflammatory factors and chemokines, leading to further NETs formation. Ultimately, this process establishes a closed loop of NETs accumulation and sustained inflammatory injury.

Neutrophils become activated and form NETs while releasing chemokines. These chemokines synergize with NETs to promote the infiltration and activation of cytotoxic immune cells into tissues and organs, representing another critical mechanism of NETs-mediated inflammatory injury (43). Neutrophils are capable of producing a diverse array of chemokines, including CCL2, CCL3, CCL4, CXCL8, CXCL1, CXCL9, and CXCL10, which primarily mediate the chemotaxis of neutrophils, monocyte-macrophages, dendritic cells, and other immune cells to sites of inflammatory injury (44, 45). In both patients with lupus nephritis and mouse models, it has been demonstrated that the kidney can induce the infiltration of neutrophils, monocytes, macrophages, T cells, and B lymphocytes, thereby causing tissue inflammatory damage through the expression of various chemokines (46). In our study, analysis of kidney tissue sequencing data using RNA-Seq confirmed the upregulation of multiple chemotactic molecules in MRL/lpr mice. Additionally, real-time PCR revealed increased expression of CCL2 in the kidneys of MRL/lpr mice as well as *in vitro* neutrophil model samples. Recent studies indicate that CCL2 plays a critical role in the chemotaxis of macrophages and neutrophils in lupus nephritis kidneys (47, 48). In infectious or traumatic diseases, the activation of NETs on other immune cells, including neutrophils, is mainly to increase the cytotoxic ability of immune cells such as phagocytosis and killing (49). Research has demonstrated that the DNA component of NETs activates and polarizes proinflammatory macrophages via TLR9/NF-κB signaling pathways (20). Moreover, NETs containing characteristic granule proteins, such as MPO and HMGB1, can stimulate plasmacytoid dendritic cells (pDCs) to produce antiviral factors (50). The interaction between NETs and T cells, mediated by T cell receptors, lowers the activation threshold of T cells and strengthens specific immune responses (51). Additional studies have confirmed that NETs facilitate T cell recruitment and promote the secretion of cytokines such as TNF, IFN-γ, and IL-6 (52, 53). In addition, the co-culture of exogenous NETs with immune cells can activate Th17 cells and induce inflammatory responses, thereby affecting immune regulation (54). In a recent study on lupus nephritis, the infusion of NETs exacerbated disease progression in a murine model of LN, accompanied by a marked reduction in the number of immunosuppressive regulatory T cells (Tregs). Conversely, treatment with DNase I restored Treg levels and mitigated disease symptoms (28). These findings are consistent with our observations: the numbers of CD4+Foxp3+CD25+ Tregs were significantly diminished in the spleen of MRL/lpr mouse models but were restored following DNase I treatment. In conclusion, chemokines and NETs released in the microenvironment of inflammation-damaged tissues in LN can further intensify inflammation and tissue injury by recruiting and activating cytotoxic immune cells.

More and more evidence indicates that NETs are formed in acute kidney injury, diabetic nephropathy, lupus nephritis, and other diseases, mediating inflammatory damage to the kidney (23, 55, 56). As a classical molecule essential for citrullination during NETs formation, peptidylarginine deiminase 4 (PADI4) is highly expressed in neutrophils and plays a critical role in the pathogenesis of SLE and LN (57). Our study further confirmed that PADI4 was significantly activated in both LN animal models and cellular models, demonstrating a close association with NETs formation. In both acute kidney injury and lupus mouse models, renal neutrophil infiltration and NETs production were markedly reduced, and renal function was significantly improved in PADI4 knockout mice (55, 58). Consequently, PADI4 serves not only as a molecular marker of renal inflammatory injury but also as a key therapeutic target for targeting NETs in the treatment of kidney diseases. In our study, in addition to PADI4, several key markers, including HMGB1, TLR4, and MYD88, were identified as being activated in the kidney following inflammatory injury. These molecular markers are not only highly expressed in neutrophils from the peripheral blood of LN patients and *in vitro* cell models but are also activated in LN animal models *in vivo*. Furthermore, their expression levels exhibit a significant upward trend with disease progression. Additionally, these markers are positively correlated with the formation of NETs in the kidney; however, the underlying mechanism requires further investigation. Nonetheless, these findings corroborate the recently reported strong association between HMGB1 (59–61), TLR4 (20, 62), and MYD88 (63, 64) and the formation of NETs as well as inflammatory damage in other organs and tissues. Moreover, they establish HMGB1, TLR4, and MYD88 as novel molecular markers for NETs-mediated renal inflammatory damage in lupus nephritis, providing new targets for the prevention, diagnosis, and treatment of this condition.

## 5 Conclusions

In conclusion, NETs activated and formed by neutrophils play a critical role in the inflammatory injury process observed in the kidneys of LN mice. In this study, DNase I, a widely recognized NETs inhibitor utilized in research, was employed to suppress NETs production in the kidneys of LN mice, thereby alleviating renal inflammatory damage. Through RNA-seq analysis and molecular detection techniques, we demonstrated that the NETs/TLR4/MYD88 signaling pathway promotes NETs formation and induces the infiltration of immune and inflammatory cells, such as T cells and macrophages, ultimately mediating renal inflammatory injury. Additionally, several molecular markers closely associated with NETs-induced kidney injury were identified (PADI4, HMGB1, TLR4 and MYD88). These findings may offer novel clinical prevention and treatment strategies for lupus nephritis.

## Data Availability

Sequencing data sets have been deposited in the gene expression omnibus (GEO) data repository under the accession number GSE301237.
