# Supplementary material for: DNase I alleviates renal inflammatory injury in MRL/lpr mice by inhibiting NETs formation
Source: Front Immunol. 2025 Oct 23;16:1656069. doi: 10.3389/fimmu.2025.1656069 (PMC12589076; doi:10.3389/fimmu.2025.1656069)
Supplement: Supplementary file 1 [file DataSheet1.docx]

Original blot image


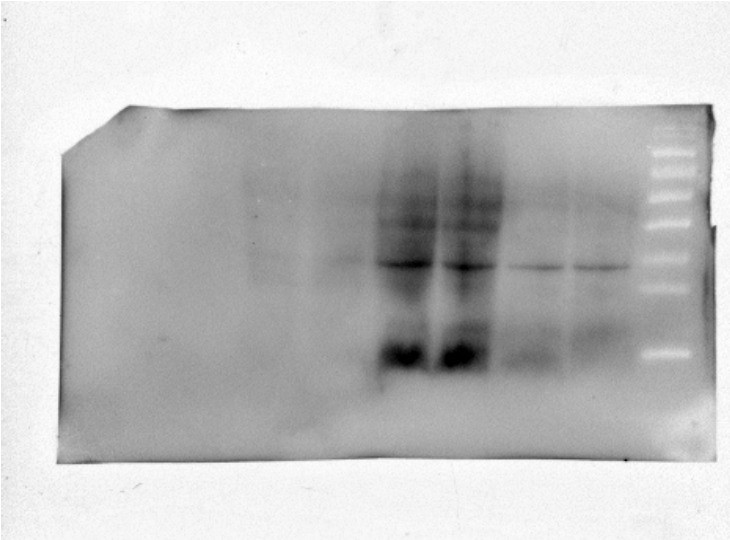


This original blot corresponds to the NE protein detected in Figure 2E.


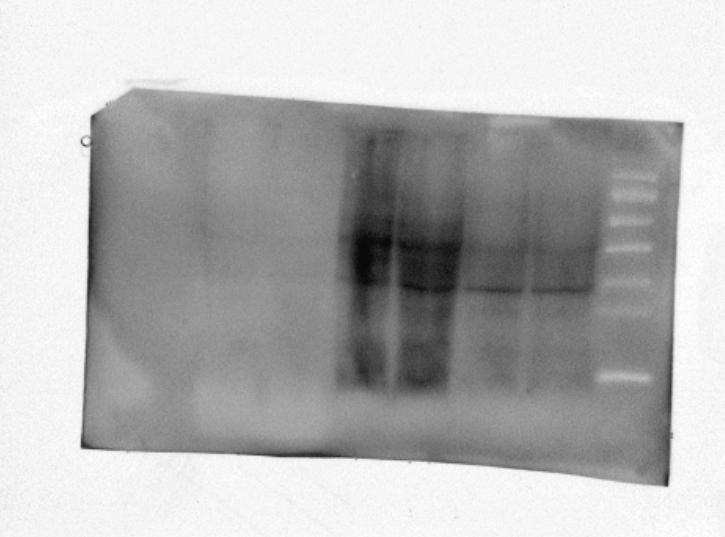


This original blot corresponds to the CitH3 protein detected in Figure 2E.


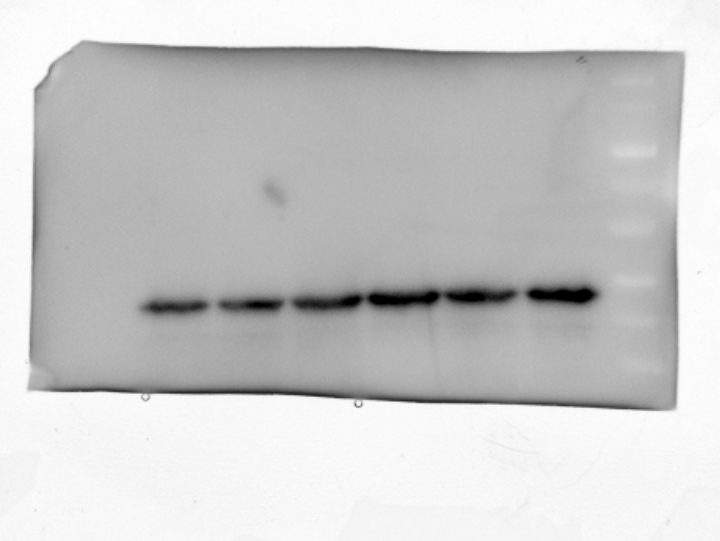


This original blot corresponds to the GAPDH protein detected in Figure 2E.


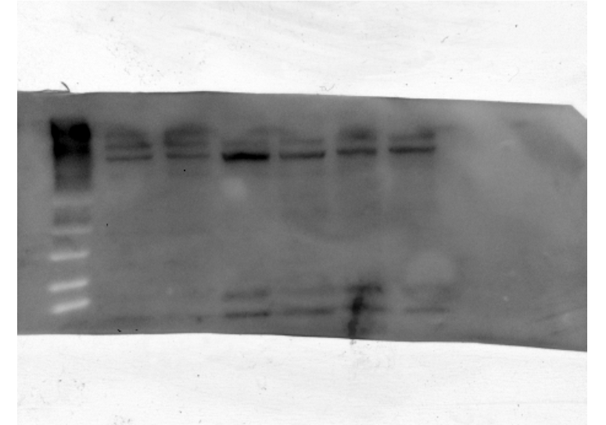


This original blot corresponds to the TLR4 protein detected in Figure 5D.


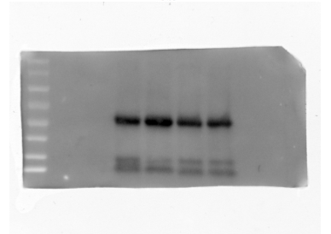


This original blot corresponds to the MYD88 protein detected in Figure 5D.


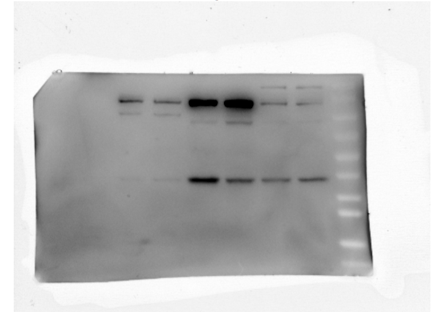


This original blot corresponds to the IL6 protein detected in Figure 5D.


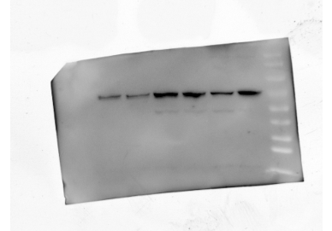


This original blot corresponds to the INOS protein detected in Figure 5D.


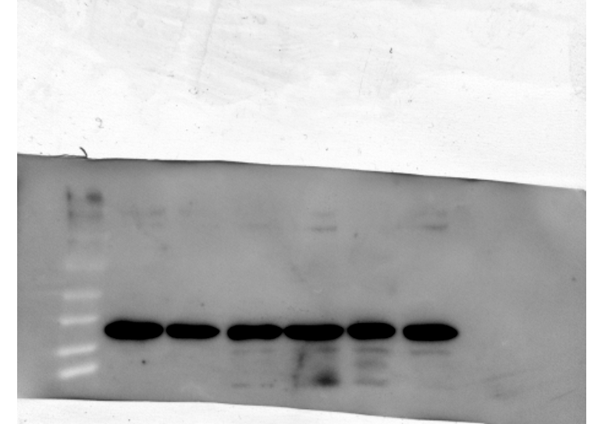


This original blot corresponds to the GAPDH protein detected in Figure 5D.
