# Supplementary material for: DNase I alleviates renal inflammatory injury in MRL/lpr mice by inhibiting NETs formation
Source: Front Immunol. 2025 Oct 23;16:1656069. doi: 10.3389/fimmu.2025.1656069 (PMC12589076; doi:10.3389/fimmu.2025.1656069)
Supplement: Supplementary file 2 [file Table1.docx]

**Table S1.** The primers used for qRT-PCR

| Gene | Sense/antisense | Fragment |  |
| --- | --- | --- | --- |
| *IL-1β* (Mouse) | 5’-CTTTCCCGTGGACCTTC-3’ | 153bp |  |
|  | 5’-CATCTCGGAGCCTGTAGTG-3’ |  |  |
| *TNF-α* (Mouse) | | 5’-GGAACTGGCAGAAGAGGCA-3’ | 119bp |
|  |  | 5’-GACAGAAGAGCGTGGTGGC-3’ |  |
| *IL-6* (Mouse) | | 5’-GTTGCCTTCTTGGGACTGAT-3’ | 185bp |
|  |  | 5’-TTGCCATTGCACAACTCTTT-3’ |  |
| *IL-8* (Mouse) | | 5’-CATTTGGGAGACCTGAGAAC-3’ | 114bp |
|  |  | 5’-CACTGGAGTCCCGTAGAAAA-3’ |  |
| *IL-17* (Mouse) | | 5’-CAATGCGGAGGGAAAGC-3’ | 159bp |
|  |  | 5’-TGGCGGACAATCGAGGC-3’ |  |
| *INOS* (Mouse) | | 5’-CTTGGGGAGACAGCGAAAT-3’ | 137bp |
|  |  | 5’-GAGGAAAGGGAGAGAGGGG-3’ |  |
| *CAPASE3* (Mouse) | | 5’-GGGACTGATGAGGAGATGGC-3’ | 127bp |
|  |  | 5’-GGGACTGGATGAACCACGAC-3’ |  |
| *CAPASE9* (Mouse) | | 5’-TGCGGTGGTGAGCAGAAAGA-3’ | 190bp |
|  |  | 5’-CTGGGAAGGTGGAGTAGGACA-3’ |  |
| *BCL2* (Mouse) | | 5’-GTGGTGGAGGAACTCTTCAGGG-3’ | 154bp |
|  |  | 5’-GCCGGTTCAGGTACTCAGTCAT-3’ |  |
| *BAX* (Mouse) | | 5’-CAGGATGCGTCCACCAAGAA-3’ | 196bp |
|  |  | 5’-CAAAGTAGAAGAGGGCAACCAC-3’ |  |
| *KIM1* (Mouse) | | 5’-ATGCCCATCTTCTGCTTGTC-3’ | 184bp |
|  |  | 5’-CCATCCAGGAATCTCCACTC-3’ |  |
| *CCL2* (Mouse) | | 5’-CACCTGCTGCTACTCATTCACC-3’ | 161bp |
|  |  | 5’-ATGTCTGGACCCATTCCTTCTT-3’ |  |
| *TLR4* (Mouse) | | 5’-GGCTCATTCACTCACTAACGG-3’ | 134bp |
|  |  | 5’-CAGGAGGGACCATCTTCATTT-3’ |  |
| *MYD88* (Mouse) | | 5’-TATCGCTGTTCTTGAACCCTC-3’ | 252bp |
|  |  | 5’-AGTATTTCTGGCAGTCCTCCTC-3’ |  |
| *PADI4* (Mouse) | | 5’-CCCTGTCAAGCGAGTTATGG-3’ | 170bp |
|  |  | 5’-AGTAACCGCTATTCCCGATG-3’ |  |
| *HMGB1* (Mouse) | | 5’-GCCTTCTTCTTGTTCTGTTCTG-3’  5’-CTCATAGGGCTGCTTGTCATCT-3’ | 135bp |
| *TGF-β* (Human) | | 5’-CCCACAACGAAATCTATGACAAG-3’ | 278bp |
|  |  | 5’-CAACCACTGCCGCACAACT-3’ |  |
| *IL-1β* (Human) | | 5’-AATGATGGCTTATTACAGTGGC-3’ | 263bp |
|  |  | 5’-AGATGAAGGGAAAGAAGGTGC-3’ |  |
| *TNF-α* (Human) | | 5’-CCTGGTATGAGCCCATCTATC-3’ | 297bp |
|  |  | 5’-CGAAGTGGTGGTCTTGTTGC-3’ |  |
| *IL-10* (Human) | | 5’-ACATCAGGGTGGCGACTCTAT-3’ | 202bp |
|  |  | 5’-TGGGCTTCTTTCTAAATCGTTCA-3’ |  |
|  | |  |  |
| *CCL2* (Human) | | 5’-CTTCTGTGCCTGCTGCTC-3’ | 154bp |
|  |  | 5’-TGCTGCTGGTGATTCTTCT-3’ |  |
| *PADI4* (Human) | | 5’-CCAGGTCTGAGATGGACAAAGT-3’ | 202bp |
|  |  | 5’-AGGGAGATGGTGAGGGTAATG-3’ |  |
| *HMGB1* (Human) | | 5’-AGCATAAGAAGAAGCACCCAGAT-3’ | 256bp |
|  |  | 5’-GGGCGATACTCAGAGCAGAAG-3’ |  |
| *TLR4* (Human) | | 5’-AAAGCCGAAAGGTGATTGT-3’  5’-CGTCTCCAGAAGATGTGCC-3’ | 244bp |
| *MYD88* (Human) | | 5’-CCCACCTTGAGCCTTATTTC-3’ | 248bp |
|  |  | 5’-CATCATTACAGTGACTCATCCC-3’ |  |
